# Molecular insights into the human ABCB6 transporter

**DOI:** 10.1038/s41421-021-00284-z

**Published:** 2021-07-27

**Authors:** Guangyuan Song, Sensen Zhang, Mengqi Tian, Laixing Zhang, Runyu Guo, Wei Zhuo, Maojun Yang

**Affiliations:** Ministry of Education Key Laboratory of Protein Science, Tsinghua-Peking Center for Life Sciences, Beijing Advanced Innovation Center for Structural Biology, School of Life Sciences, Tsinghua University, Beijing, China

**Keywords:** Cryoelectron microscopy, Protein translocation

## Abstract

ABCB6 plays a crucial role in energy-dependent porphyrin transport, drug resistance, toxic metal resistance, porphyrin biosynthesis, protection against stress, and encoding a blood group system Langereis antigen. However, the mechanism underlying porphyrin transport is still unclear. Here, we determined the cryo-electron microscopy (cryo-EM) structures of nanodisc-reconstituted human ABCB6 trapped in an apo-state and an ATP-bound state at resolutions of 3.6 and 3.5 Å, respectively. Our structures reveal a unique loop in the transmembrane domain (TMD) of ABCB6, which divides the TMD into two cavities. It restrains the access of substrates in the inward-facing state and is removed by ATP-driven conformational change. No ligand cavities were observed in the nucleotide-bound state, indicating a state following substrate release but prior to ATP hydrolysis. Structural analyses and functional characterizations suggest an “ATP-switch” model and further reveal the conformational changes of the substrate-binding pockets triggered by the ATP-driven regulation.

## Introduction

ATP-binding cassette (ABC) transporters, which transport a variety of substrates such as nutrients, drugs, and ions by hydrolyzing ATP, are a large class of transmembrane proteins existing extensively in various organisms, ranging from bacteria to human^1–3^. In bacteria, ABC transporters usually mediate the influx of essential nutrients and ions for growth, and the efflux of toxins for self-protection^4,5^. In humans, 48 kinds of ABC transporters were characterized to maintain the sophisticated physiological homeostasis^6,7^. ABC transporters share a canonical structure that contains two nucleotide-binding domains (NBDs) and two transmembrane domains (TMDs)^8^. All NBDs contain the characteristic motifs, Walker A and Walker B, which could utilize the energy of ATP to drive transmembrane transportation, while TMDs usually contain 6–12 transmembrane *α*-helices and provide a distinctive substrate-binding site^6^.

ABCB6 is an 842-amino acid protein that belongs to the B subfamily of the ABC transporter. ABCB6 is a “half-transporter” that contains a single pair of TMD and NBD, but needs to form a homodimer to be functional^8^. The ABCB6 gene was identified in 1997 as a drug-resistance gene from the human liver and was previously known as MTABC3 and P-glycoprotein-related protein (PRP)^9^. Since then, the function of ABCB6 is gradually expanded, such as drug resistance^10–12^, toxic metal resistance^13^, promoting porphyrin biosynthesis^7^, protection against stress^14^, and encoding a blood group system Langereis antigen^15^. ABCB6 is a porphyrin transporter located in the outer membrane of mitochondria that may regulate the heme biosynthetic pathway together with the plasma membrane porphyrin transporter ABCG2^16,17^. ABCB6 was also reported to appear in the plasma membrane^18^; however, a role for ABCB6 in substrate efflux across the plasma membrane has not been established.

ABCB6 is crucial in the process of heme biosynthesis^19^. Heme is a kind of porphyrin bound with ferrous iron and plays a vital role in many biological processes^20^. The ferrous iron in heme can carry oxygen, which capacitates erythrocytes to transport oxygen. In mitochondria, heme is involved in electron transfer and can regulate catalase to protect cells from the reactive oxygen species (ROS)^14^. The biosynthesis of heme is a coordinated process between cytosol and mitochondria. In the cytosol, a series of reactions can change 5-aminolevulinic (ALA) to coproporphyrinogen III (CPgen III), then CPgen III is imported into mitochondria by ABCB6^21^ to further finish the synthesis of heme. Defects of heme synthesis can affect the hematopoietic system, hepatic function, and nervous systems in humans^22^.

The molecular mechanisms of transmembrane transportation and substrate recognition for ABCB6 remain unclear, as well as the mechanism underlying pathogenic mutations and drug resistance. Recently, Wang et al.^23^ reported the cryo-EM structure of human ABCB6 and provided a structural glimpse of this transporter. However, this study mainly bases on a 4.0-Å resolution model, which is borderline sufficient to identify side chains. Consequently, the resultant biased model has impeded the identification of the unique loop and cavities within the TMD, which is indispensable in substrate translocation and will be discussed below. Here, we present the structures of human ABCB6 (hABCB6) in apo- and nucleotide-bound states and reveal a unique “plug” and two cavities essential for substrate transport using single-particle cryo-EM. These new structures allow us to propose an “ATP switch” model^24^, by which the plug and two cavities undergo conformational changes to facilitate substrate translocation. Our results provide a framework for understanding the mechanism of transmembrane transportation and for structure-based drug design.

## Results

### Structural determination and the overall inward-facing structure of hABCB6

**Fig. 1:**
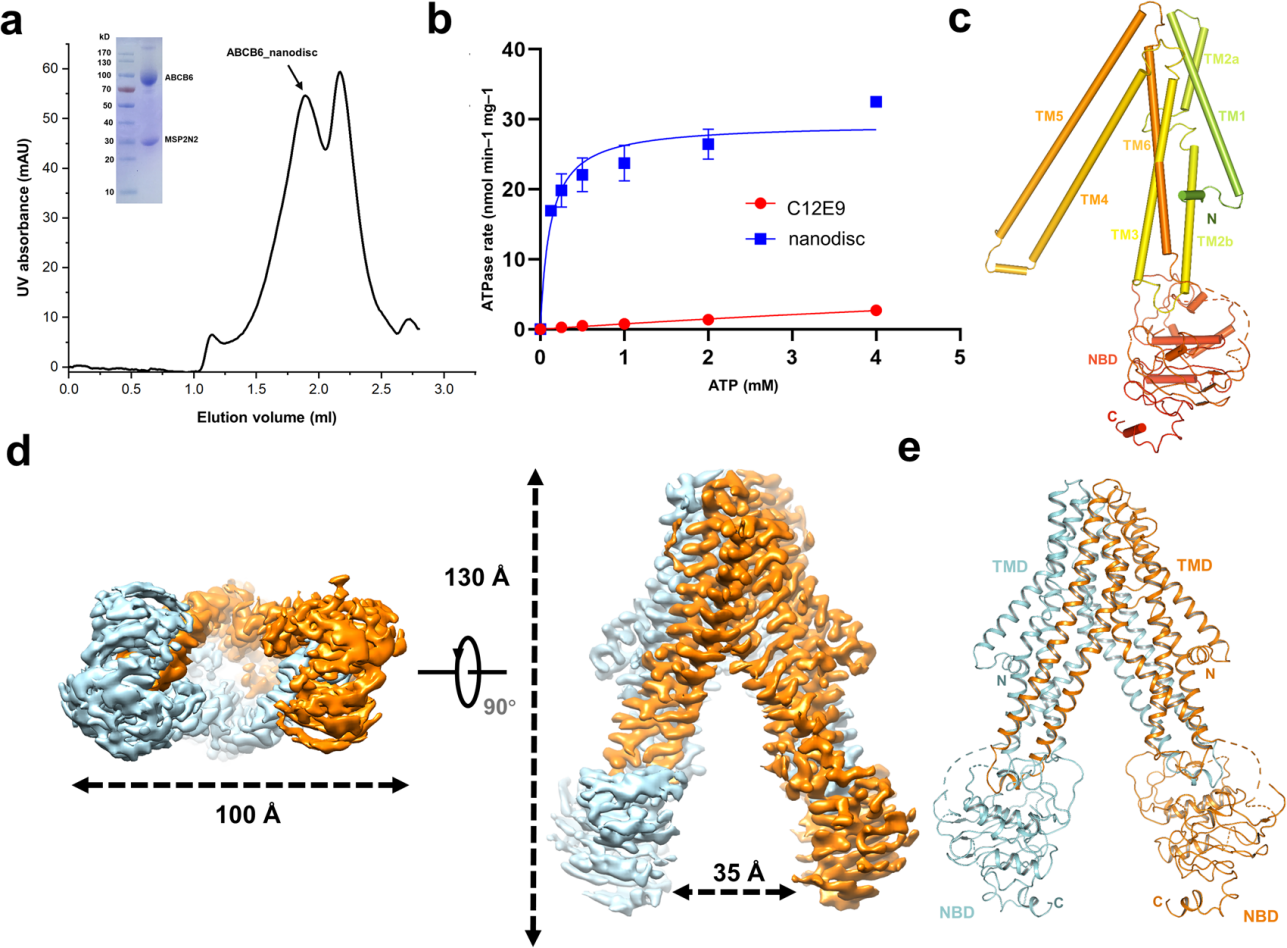
Structure of inward-facing hABCB6. **a** Size-exclusion chromatography (SEC) profile of purified WT hABCB6 reconstituted into lipid (POPC:POPE = 3:1) nanodiscs. UV absorbance (mAu), absorbance at 280 nm. Inset: SDS-PAGE analysis showing bands for hABCB6 (theoretical molecular weight (MW), 94 kDa) and the nanodisc scaffold protein MSP2N2 (theoretical MW, 30 kDa). **b** ATPase activity of hABCB6 in a lipid environment (blue squares, POPC and POPE nanodiscs; the blue curve is a fit to Michaelis–Menten kinetics) and in detergent (red circles and red curve, detergent C_12_E_9_). **c** Topology diagram of monomer hABCB6 with TMs and NBDs numbered. **d** Surface representation of apo-state hABCB6 in bottom and side views. **e** Cartoon representation of our apo-state hABCB6 model.

To gain insight into the architecture of hABCB6, we tried to purify the overexpressed hABCB6 from HEK293 cells (Fig. 1a). To test whether the recombined protein is functional, we measured the ATP activity of hABCB6 in different conditions. The hABCB6 purified within detergent C_12_E_9_ shows little ATP affinity (Fig. 1b). The low ATP affinity indicates the conformation block of hABCB6. To mimic the physiological conditions of hABCB6, we reconstituted hABCB6 into a nanodisc with a mixed lipid to imitate the mitochondrial membrane. The ATP assay of hABCB6 in a nanodisc shows a low affinity to ATP (*V*_max_ = 27–32 nmol/min/mg) (Fig. 1b), compared with the reported affinity of ABCB6 reconstructed in liposomes (*V*_max_ = 492 nmol/min/mg)^25^. These results indicate that the hABCB6 nanodisc maintains a relatively stable conformation, suggesting that the lipid environment is important for the ATP activity of hABCB6, and that our recombined nanodisc hABCB6 is suitable for structural studies.

We solved the apo-state cryo-EM structure of human hABCB6 in nanodiscs at the resolution of 3.6 Å according to the Fourier shell correlation (FSC) 0.143 criterion (Supplementary Fig. S1 and Table S1). The overall structure of apo-state hABCB6 is similar to most eukaryotic ABC transporters and some bacterial exporters that belong to B-family ABC exporters^1^. hABCB6 is a half-transporter consisting of one NBD and one TMD (Fig. 1c). The TMD contains six transmembrane helices, among which TM4 and TM5 insert into the other hABCB6 monomer in the homodimeric transporter. The hABCB6 apo-state structure shows an inward-facing conformation due to the absence of ATP (Fig. 1d, e). The overall structure shows an elongated shape with 130 Å in height and the two NBDs, like two “legs”, are separated by a distance of 35 Å (Fig. 1d). The NBD has a lower local resolution than TMD in apo-state (Supplementary Fig. S1e), but an accessible crystal structure of hABCB6 NBD can be docked into the map^26^.

The interface between two hABCB6 monomers shows key amino acids to stabilize it. A feature of B-family exporter, like Type Ι ABC transporter^1^, is the transmembrane domain-swapped conformation between the two monomers (Fig. 1e). In hABCB6, the TM4/5 from one monomer interacts with NBD and TM2 from another monomer (Supplementary Fig. S2). Triggered by ATP binding, the closure of the opposing NBD will alter these interactions and propagate the conformational changes to the TMD, thus facilitating the substrate translocation.

The N-terminal TMD0 is a unique domain found in several ABC transporters: five multidrug-resistance proteins (MRP1/2/3/6/7)^27^, SUR1/2^28,29^, and TAP^30^. TMD0 is known to function as a linker domain to help ABC transporters interact with other proteins^29^. hABCB6 has a TMD0, ranging from amino acid 1 to 244, but is obscure in the map due to its flexible conformation. Until now there are no interacting proteins found with the TMD0 of hABCB6; thus further studies are needed to find such proteins to stabilize TMD0 domains before obtaining the structure of TMD0.

### The unusual substrate-binding site of hABCB6

**Fig. 2:**
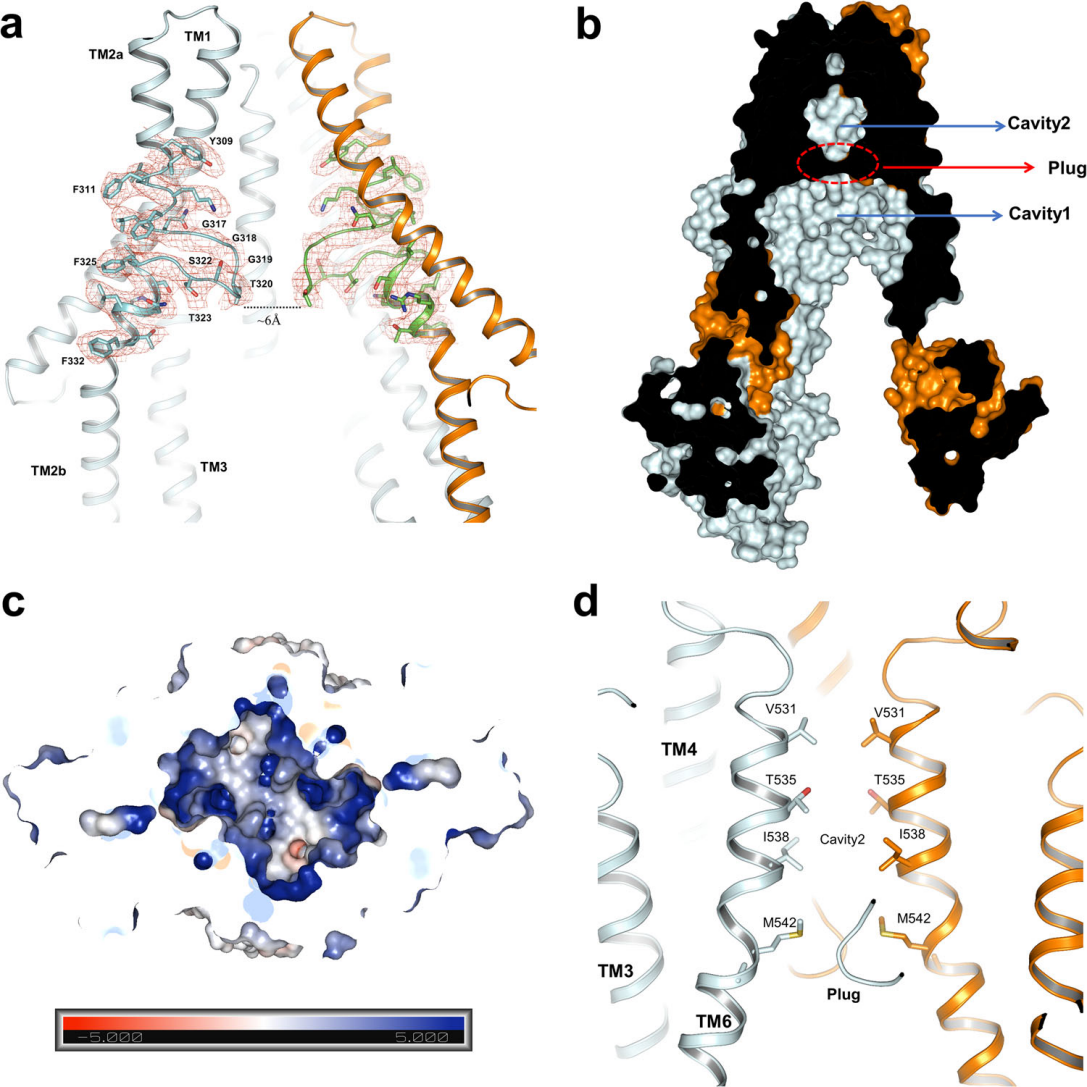
Substrate-binding site and translocation pathway. **a** Cartoon representation of the plug within the TMD of hABCB6. The EM density of the plug and neighboring residues is shown at a contour level of 8*σ*. The surrounding residues are shown as sticks and labeled. **b** Sagittal slice through a surface representation of the hABCB6 TMD with cavities 1 and 2 indicated and the “plug” depicted by a red dashed circle. **c** Electrostatic surface potential of cavity 1 viewed from the cytoplasm with NBDs removed. Scale: red, negative (−5 kT/e); blue, positive (+5 kT/e). **d** Cartoon representation of cavity 2 at a vertical view of “plug”, colored as in panel **a**. The related residues are shown as sticks and labeled.

In apo-state hABCB6, we identified a special TMD region that shares low-sequence conservation with other ABC transporters (Supplementary Fig. S3). The TMD region of hABCB6 contains a loop “plug”, just like a gate, which includes a sequence of “GGGTGSTG” in the middle region of TMD (Fig. 2a). The “plug” intervening TM2a and TM2b from one monomer interacts with TM5 and TM6 from another monomer of hABCB6 (Supplementary Fig. S2b), indicating that the “plug” is important for maintaining apo-form conformation and may undergo conformational changes during the transport process.

Previously Atm1/ABCB7/HMT1/ABCB6 subfamily model built by NaAtm shows that apo-form contains only one cavity for substrate binding^31,32^. In the apo-state, hABCB6 adopts an inward-facing conformation and TMDs of hABCB6 contain the substrate-binding cavity which is divided into two parts: a closed cavity 2 and an open-form cavity 1 (Fig. 2b), different from the consecutive substrate-binding pocket found in most ABC transporters. The distance between those two “plugs” is about 6 Å (Fig. 2a) and it is impassable for the substrates such as porphyrin. This kind of “plug” has been reported in another heme transporter ABCG2 that only contains two L–L bumps, which function as a “checkpoint” during the transport process^33–35^. The “plug” of hABCB6 comprises consecutive flexible residues that confer this long loop contributing a flexible pocket suitable for multi-substrate binding. Consistent with the “L-L plug” in ABCG2^33–35^, our structure suggests that the “plug” in the inward-facing conformation has a function of obstructing substrates and could be opened during ATP-driven conformational change in hABCB6.

In the resolved ABC transporters, MRP1 has a positively charged “P-pocket” and a hydrophobic “H-pocket” to bind GSH that contains both hydrophobic compounds and a negatively charged residue^36^, whereas CFTR only forms a positively charged cavity for the accommodation of negatively charged substrates^37^. In our structure, the “plug” together with the neighboring residue W546, provides a hydrophobic environment for substrate translocation (Supplementary Fig. S4a). The “plug” and W546 are highly conserved in ABCB6 among different species (Supplementary Fig. S5). We proposed that the strategically positioned “plug” and W546 may play special roles in the function of hABCB6. Similar to the “H–P” binding pocket of MRP1^36^, an open pocket with a hydrophobic and positive charged environment was observed in hABCB6. This pocket facilitates the binding of porphyrin (Fig. 2c and Supplementary Fig. S4b–d), such as Protoporphyrin IX (PPIX), which contains both anionic carboxylate side chains and the tetrapyrrole moiety (Supplementary Fig. S4d), and is considered as a substrate of ABCB6^19^. On the opposite side, cavity 2 of hABCB6 is a narrow funnel that is suitable for applanate substrate (e.g., porphyrin) transport (Fig. 2d). The “plug” divides TMD of hABCB6 into two pockets and it has interactions with the neighboring helices, contributing to the stability of the “plug” itself and apo-state conformation. Based on the structure, cavity 1 of hABCB6 seems to be vital in substrate recruitment, and cavity 2 is responsible for the transport of the substrate.

### The occluded conformation of hABCB6

**Fig. 3:**
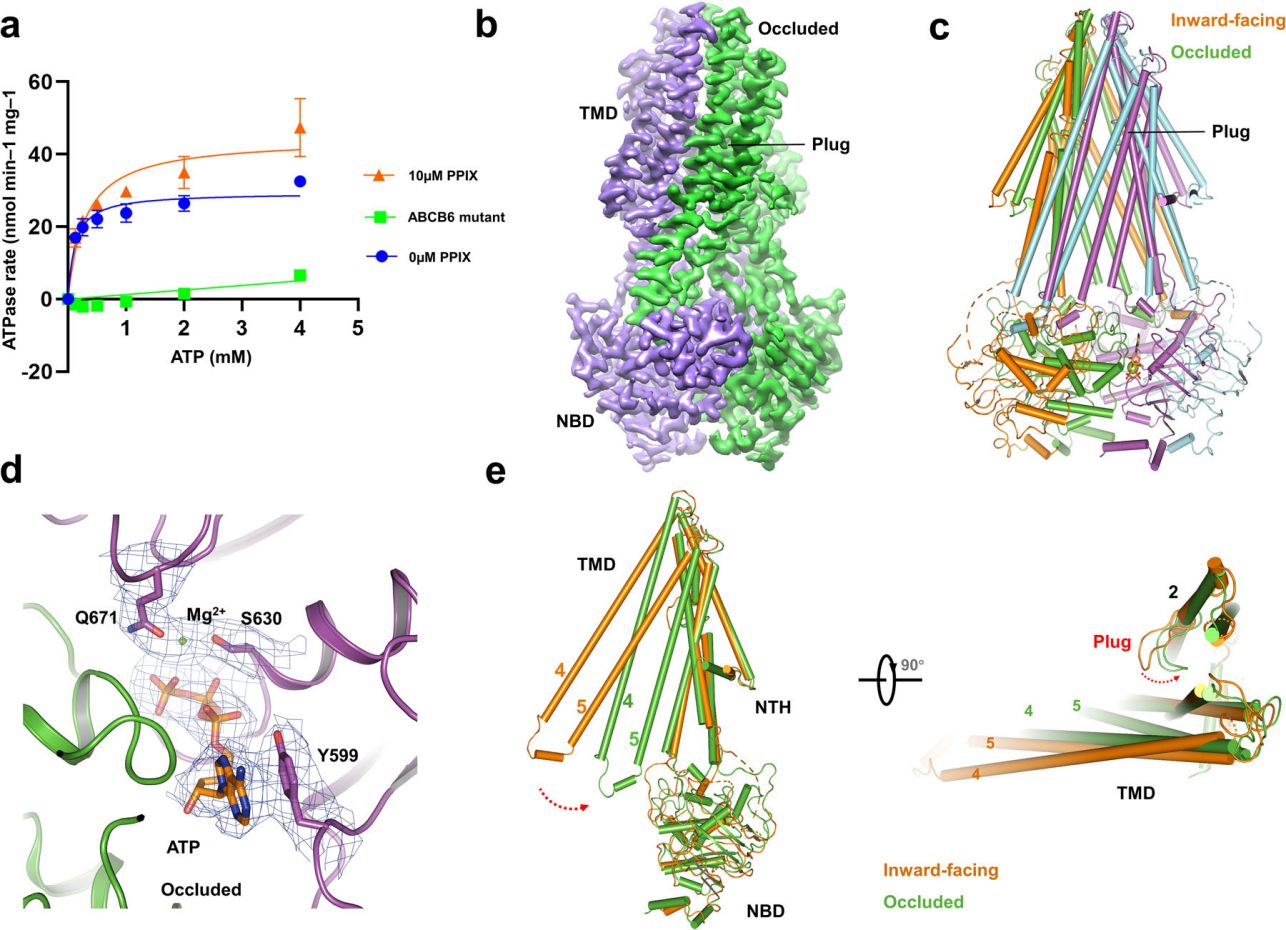
Occluded conformation of hABCB6. **a** ATPase activity of WT hABCB6 in lipid nanodiscs with different concentrations of PPIX: 0 μM (blue circles, blue curve) and 10 μM (red triangles and red curve). ATPase activity of hABCB6[EQ] in a nanodisc is shown as green squares and green curve. Data points indicate means of three independent measurements and error bars indicate SD. **b** Surface representation of occluded conformation. **c** Stick model comparison between the occluded and inward-facing structures. **d** Nucleotide-binding site and electron microscopy density of hABCB6, ATP, Mg^2+^, and residues around ATP are labeled. **e** Stick model comparison between the occluded and inward-facing monomer conformation. The swap direction of TM4, 5, and “plug” is labeled by a red arrow.

To elaborate on the detailed transport mechanism of hABCB6, we sought to obtain a nucleotide-bound conformation to illustrate the conformational changes during substrate transport. To this end, we overexpressed the hABCB6 E752Q mutant (hereafter referred to as hABCB6[EQ]), which harbors an E752Q mutation located in the Walker B motif and was suggested to arrest the transporter in an occluded conformation in the presence of ATP based on sequence conservation^38^ (Supplementary Fig. S3). The wild-type hABCB6 shows a substrate-stimulated ATPase activity following the addition of PPIX, while the hABCB6[EQ] mutant reveals little-to-no ATPase activity, largely due to the deficiency of ATP hydrolysis (Fig. 3a).

Furthermore, we solved the nucleotide-bound structure of human hABCB6[EQ] at 3.5-Å resolution by using cryo-EM (Fig. 3b, Supplementary Fig. S6 and Table S1), allowing for unambiguous side-chain placement for most of the protein (Supplementary Fig. S7). The nucleotide-bound hABCB6[EQ] forms an occluded conformation compared with the apo-state inward-facing conformation (Fig. 3c). The remarkable change between nucleotide-bound state and apo-state is the closure of opposing NBDs triggered by the binding of ATP and Mg^2+^ (Fig. 3d). The closure of the two NBDs leads to an anticlockwise rotation of TM4 and 5, which is a common feature of conformational change within ABC transporters (Fig. 3e).

**Fig. 4:**
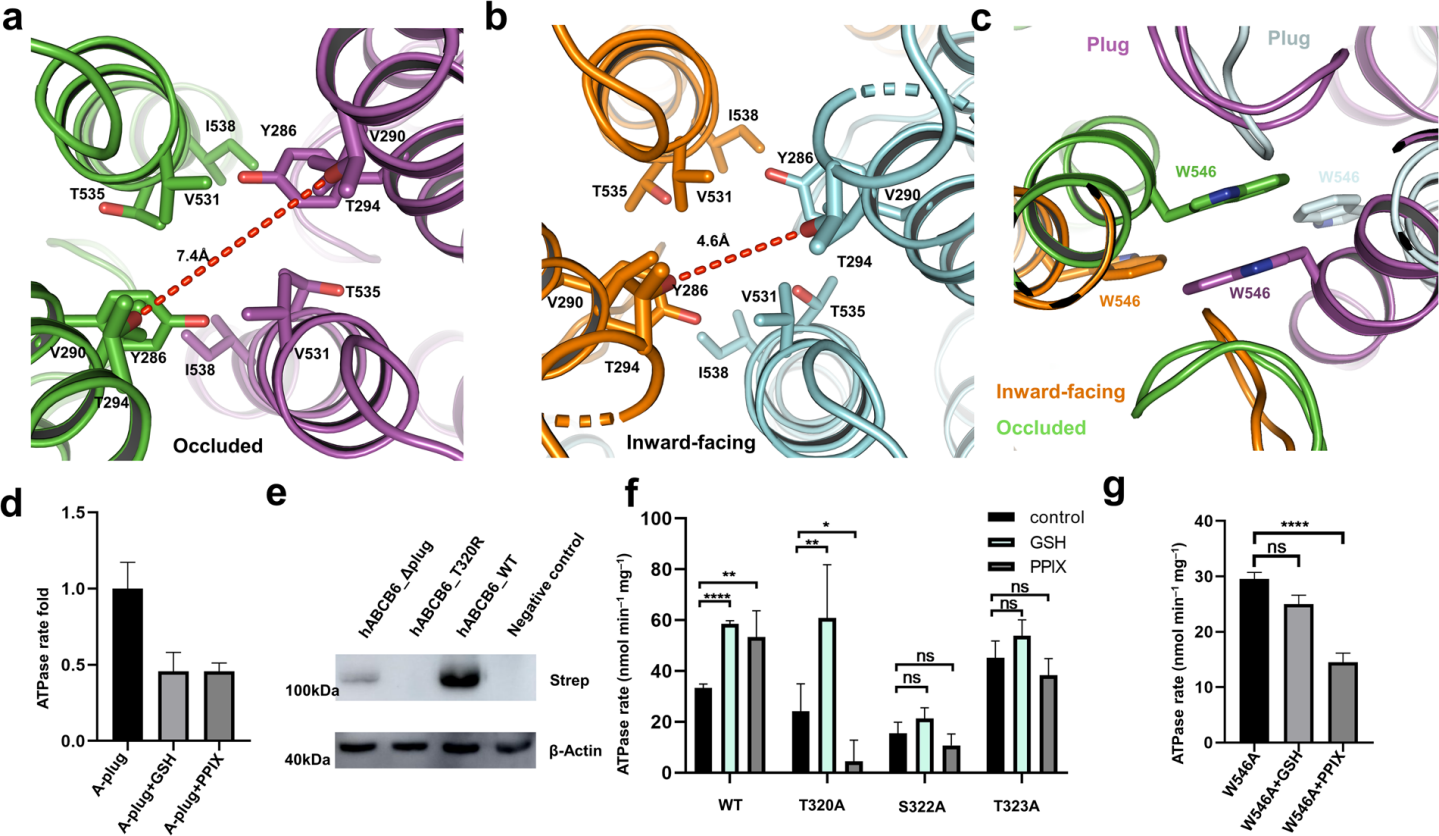
Comparison between the inward-facing and occluded conformations. **a** Cavity 2 in occluded conformation. **b** Cavity 2 in inward-facing conformations. **c** The entrance of cavity 2 in inward-facing and occluded conformation. **d** The total expression levels of the wild-type, T320R, and plug-truncated form (residues 317–324 (GGGTGSTG) deleted) in HEK293F cells with actin as the loading control. The hABCB6 proteins carry a strep tag. **e** ATPase activity of hABCB6-WT, T320A, S322A, and T323A mutants with 4 mM ATP in the presence of 35 mM GSH or 10 μM PPIX. Error bars represent SD, *n* = 5. **f** ATPase activity of A-plug with 4 mM ATP in the presence of 35 mM GSH or 10 μM PPIX. Error bars represent SD, *n* = 5. **g** ATPase activity of W546A mutant with 4 mM ATP in the presence of 35 mM GSH or 10 μM PPIX. Error bars represent SD, *n* = 5.

In the lipid bilayer, the closure of the two NBDs also triggers the rotation of the plug to eliminate the barrier for substrate translocation (Fig. 3e) and the TMDs adopt a collapsed conformation without substrate binding. In the inward-facing conformation, the “plug” within the middle of the TMD region, and the upper constriction sites containing residues Y286 and V531, together forbid the access of substrate translocation (Fig. 4a, b, and Supplementary Fig. S8). The differences between these two conformations in the TMD are the decreases of cavities 1 and 2 in the occluded conformation, as well as a slight opening of the extracellular vestibule (Fig. 4a, b, and Supplementary Fig. S8). The occluded hABCB6 is similar to the outward-facing ABCB1, but the extracellular vestibule of hABCB6 is still more closed than the outward-facing ABCB1. The occluded hABCB6 also shows a smaller opening aperture at cavity 2 than the outward-facing conformation of Sav1866 (Supplementary Fig. S9a, b). The extracellular vestibule of cavity2 in inward-facing hABCB6 is not fully closed compared to the extracellular part of inward-facing ABCB1 (Supplementary Fig. S9c). Those findings indicate that the “plugs” maintain the impermeability in inward-facing conformation. In the process of removing the barrier by changing the conformation of the “plug”, cavity 1 in the apo-state is completely closed off from the intracellular matrix, and the “plug” rotates to the other side to create a pathway connecting to cavity 2. The extracellular vestibule of cavity 2 is opened following the separation of T294 (Fig. 4a, b), and the steric clash pushes substrates through cavity 2 and contributes to the substrate expulsion.

In the apo-state, the “plug” in TMD not only plays a role as a barrier to forbid the entry of substrates to cavity 2, but also maintains interactions with the neighboring monomer. In the nucleotide-bound structure, the “plug” swinging to TM6 belongs to the same monomer to form an open state and clears a pathway toward the outside, accompanied by the conformational change of the two W546 residues and forming π–π interaction to further constitute a “bottom” of the cavity 2 (Fig. 4c). In this process, the “H–P” cavity 1 disappears and cavity 2 is decreased to extrude substrates. Our nucleotide-bound structure reveals a substrate-free conformation, despite the incubation of 20 μM PPIX with the protein before cryo-EM sample preparation. The structure indicates that cavity 2 of hABCB6 is not suitable for the accommodation of substrates in this state and substrates escaped from this cavity before the hydrolysis of ATP. This structure is consistent with the “ATP-switch” model^24^ and helps to elucidate the transport mechanism of hABCB6.

### Substrate binding and recognition of hABCB6

Glutathione (GSH) is essential for the Fe/S protein biogenesis and cellular iron regulation, and has been identified as a substrate for efficient ATPase activity of the yeast mitochondrial ABC transporter Atm1^39,40^. Given that Atm1 and ABCB6 belong to the same Atm1/ABCB7/HMT1/ABCB6 ortholog^41,42^, and that Wang et al.^23^ reported hABCB6 has the GSH-conjugated ATPase stimulation, so we proceeded to investigate the possible functional role of GSH in the translocation of hABCB6. The ATPase rate of hABCB6 was significantly increased following the addition of GSH, featuring the functional role of GSH as a substrate of hABCB6 transporter, reminiscent of the established hABCB6 substrate PPIX (Fig. 4d, e). To validate the importance of this specific “plug” in substrate binding and recognition, we performed mutagenesis (T320R) and truncation (residues 317–324 (GGGTGSTG) deleted) of this plug. The expression levels of the mutation and truncation forms were largely impaired compared with the wild-type protein (Fig. 4d), highlighting the indispensable role of this unique “plug” in hABCB6. To test the importance of this “plug” in transporter activity, we replaced residues 317–324 (GGGTGSTG) with alanine (hereafter referred to as A-plug mutant). The resultant A-plug mutant displayed an inhibitory effect following the addition of PPIX or GSH on ATPase activity (Fig. 4f).

To further explore the residues for substrate binding, we performed mutagenesis of residues T320, S322, T323, and W546 to analyze ATPase rate. Compared with the wild-type hABCB6, T320A mutant retains the GSH-stimulated ATPase activity, which indicates that T320 is not involved in the direct binding of GSH; the PPIX inhibitory effect of T320A mutant is not expected to manifest since the T320A mutant turns the substrate into an inhibitor (Fig. 4e), implying the pivotal role of T320 in PPIX translocation process. Such a reciprocal difference between the substrates GSH and PPIX on T320A mutant indicates that residue T320 is involved in the recognition of these two ligands. On the other hand, S322A and T323A mutations abolish both the GSH- and PPIX-stimulated ATPase activity (Fig. 4e), which reveals that S322 and T323 are key residues for substrate binding. Together, these mutagenesis results of the “plug” emphasize the essential role of the “plug” in substrate binding and recognition. Of additional importance at the hydrophobic cavity, W546A mutant lost the GSH-stimulated ATPase activity, indicating that residue W546 is important for GSH binding, whereas W546A showed a significant difference in ATPase activity with and without PPIX (Fig. 4g). Residue W546 is essential for the translocation of PPIX, akin to that of T320 in PPIX translocation.

Our structures provide a foundation to explore the substrate-binding sites. To this end, we tried to dock the ligands such as PPIX and GSH to our inward-facing structure to reveal the possible substrate-binding sites (Supplementary Fig. S10a, b). To verify the function of those residues, we separately mutate the interacting residues and performed the substrate-stimulated ATPase assay. The resultant PPIX-related residue mutants (T432A and N498A) and GSH-related residue mutants (R276A, R330A, and T394A) all lost the substrate-stimulated ATPase activity (Supplementary Fig. S10c, d).

### Structural interpretation of the pathogenic mutations

Several missense mutations of hABCB6 were reported in hereditary diseases (Supplementary Fig. S11a). Those mutations involve substrate-binding sites, ATP-binding sites, and some might affect transporter stability. As a porphyrin transporter, hABCB6 is described as a genetic modifier of hereditary porphyria in previous reports^19,21^. The investigation of rare-variant alleles of hABCB6 in porphyria patients shows that A492T, G588S, A681T, R192Q, R276W, and T521S contribute to the porphyria phenotype. In our structure, R192 is missing due to the low resolution of the flexible region; G588S and A681T are in the NBD; R276W and A492T are in the TMD; T521S locates in the “top” of protein near the outer side of the membrane. All of those mutations lead to the functional defects of hABCB6, but they are not located in substrate-binding sites. Although A492T is in the transmembrane domain, it can impair the ATP-binding ability of hABCB6^43^, which indicates that A492T can affect the conformation of hABCB6. In our structure, T521S and A492T both locate in the turn of main helices and interact with residues around them to stabilize or maintain the conformation of the helix bone and finally, affect the ATP coupling efficiency. Similarly, R276W could break the interaction between R276 and D397, and lead to an unstable conformation (Supplementary Fig. S11b). A681T has interactions with amino acid arrangement at the interface between NBD and TM4/5, and may affect the stability of the homodimer^43^.

In addition to hereditary porphyria, mutations of hABCB6 also cause dyschromatosis universalis hereditaria (DUH)^44^, ocular coloboma^45^, and familial pseudohyperkalemia^46,47^, and lead to the non-Langereis antigen. Some mutations can lead to the defect of eyes and pigmentation, such as A57T, L811V, and Q555K^48^, which indicates that hABCB6 has unrevealed functions other than porphyrin transport. In the substrate-binding pocket, the long sequence of amino acids GGGTGSTG may contribute to a broad spectrum of substrate combinations. The mutation S322R in DUH locates within the substrate-binding pocket, but the phenotype appears normal in respect of porphyrin biosynthesis^44^, indicating that hABCB6 can probably transport some unknown substrates other than porphyrin. Other mutations in familial pseudohyperkalemia, R357Q/W, R723Q, R276W, and V454A, lead to the phenomenon of potassium ion leakage through the erythrocyte membrane^49^. Although hABCB6 is a homolog of HMT that transports heavy metal ions^13^, the mechanism of K^+^ leakage, which might be caused by the incompletely closed inward-facing conformation, still needs to be explored.

## Discussion

Two ABC transporters, ABCB6 and ABCG2, are mainly involved in the heme biosynthesis^14,16,50^. Different from a compact cassette ABCG2, our structure shows a spindly inward-facing conformation in an apo-state, and it contains a big substrate-binding pocket similar to MsbA that transports a larger molecular lipid A-core^51^. Both ABCB6 and ABCG2 have two cavities, largely due to their broad-spectrum substrates. In ABCG2, cavity 1 is exposed to the cytoplasm and the inner leaflet of the plasma membrane can help to recruit substrate. We propose that cavity 1 in hABCB6 may play similar roles in substrate recruitment. Our structure shows an “H + P” pocket, consisting of the “plug” and amino acids around cavity 1, to recruit substrates with both hydrophobic and negatively charged properties. Our structure also shows differences with the previous hABCB6 structure^23^. The lower-resolution structure of the reported hABCB6 impeded the identification of the “plug” and thus it proposes a “plug” consisting of Y286 and V531, among which no obvious conformational changes were observed in our inward-facing and occluded structures.

Although there are many ramifications of porphyrin being substrates of hABCB6, the efficiency of transport is different among them^25^. It is plausible that hABCB6 comprises an extra module to recruit and identify substrates, through which the ligands should accommodate the environment of cavity 1, and then ligands in cavity 1 touch the “plug” to confirm whether it is allowed to access. If the ligand can be recognized by the “plug”, the “plug” changes conformation and becomes the entrance of cavity 2 after ATP binding. Different from the small size of cavity 2 in TMD of ABCG2^52^, cavity 2 in hABCB6 is larger, which is adaptable for porphyrin transport. But the slight bulk change of cavity 2 between those two conformations is unexpected (Fig. 4a, b), which means that cavity 2 in inward-facing conformation is similar to that in the occluded state. This phenomenon demonstrates a sequential order of hABCB6 conformation change driven by ATP and the elimination of the “plug”. It also reveals the important roles of the “plug” in the transport cycle of hABCB6.

**Fig. 5:**
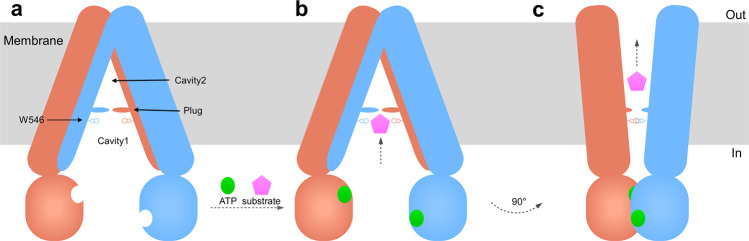
Proposed transport mechanism. **a** An inward-facing model with cavity 1, 2, W546, and “plug” labeled. **b** Substrate (pink pentagon) binds to protein and cannot access cavity 2 due to the “plug”. In the “substrate-binding” conformation, ATP (green circle) binds to NBD and dimerized NBDs. **c** ATP-driven conformation change in hABCB6 occurs owing to the closure of the NBD interface, which further converts the transporter to an outward-facing conformation that releases substrate to the outside. **a** and **c** indicate the conformations as apo-state and nucleotide-bound hABCB6, respectively.

According to our structures, we propose a mechanism of hABCB6 to identify and transport porphyrin (Fig. 5). Considering the difference in TMDs between ABCB6 and ABCB1, there are still some adaptations of the hABCB6 transport mechanism, especially the substrates binding and ATP-triggered transport pathway change. We deduce that the long hydrophobic “plug” coordinating with W546 can help to form an adaptable cavity for the binding of porphyrin. After the porphyrin binding to the pocket, which makes porphyrin to be recognized by the “plug”. After binding substrates, the helices around the cavity interact with substrates; the “plug” decreases some of the interactions between the helix around it, and hABCB6 changes to ATP-sensitive conformation and excludes the “plug” via the ATP-driving conformation change. Then the substrates move to cavity 2 and lose the high-efficiency combination to hABCB6. The final step for the transport process is substrate releasing and the subsequent structural transition back to the inward-facing conformation.

Collectively, our inward-facing conformation structure shows a distinct “plug” and two cavities that are different from other ABC transporters, including the recently reported inward-facing hABCB6. Our two structures for the first time reveal the “plug” switch in hABCB6 and provide an idea about how the ABC transporter changes the pocket into different properties to transport substrates. This model also delineates an outline of the porphyrin transport cycle. Moreover, hABCB6 is related to resistance to anti-cancer drug such as SN-38 and vincristine^10^. Structual information of the special region in TMD of hABCB6 can help to design inhibitors or drugs.

## Materials and methods

### Cell culture and transfection

Plasmids encoding *Homo sapiens* ABCB6 were kind gifts from Professor Jiahuai Han (the Xiamen University, China). For protein expression, *Homo sapiens* ABCB6 was cloned in-frame with a C-terminal Strep-tag into the pcDNA3.1 expression vector. The ABCB6 mutants were generated with a standard two-step PCR-based strategy. HEK293F cells were cultured in SMM 293T-I medium (Sino Biological Inc.) under 8% CO_2_ in a Mulititron-Pro Shaker (Infors, 120 rpm) at 37 °C. When the cell density reached 2 × 10^6^ cells per ml, for the transfection of ABCB6 and ABCB6 mutant, the cell was transfected with the mix of plasmid and PEI (plasmid:PEI:cell = 1mg:3mg:1L). The transfected cells were cultured for 48 h before harvest. HEK293F cells (R79007) were purchased from Thermo Fisher Scientific Inc., and all experiments were performed in compliance with the institutional guidelines of Tsinghua University.

### Protein expression and purification

For the protein purification of the ABCB6, two liters of transfected HEK293F cells were harvested by centrifugation at 3000× g. Cell pellets were resuspended in lysis buffer containing 20 mM Hepes, pH 7.4, and 150 mM NaCl, a protease inhibitor cocktail tablet (Roche, 1:1000), and lysed by sonication for 5 min. The cell membrane was pelleted after a 100,000× g ultracentrifugation for 1 h. The membrane was resuspended in a buffer containing 20 mM Hepes, pH 7.4, 150 mM NaCl, 2 mM DTT, and 1% (w/v) C_12_E_9_ for 1.5 h with gentle rotation at 4 °C. After ultracentrifugation at 100,000× g for 15 min, the supernatant was incubated with Strep-Tactin Sepharose (IBA) for 0.5 h with gentle rotation at 4 °C. The resin was washed extensively with wash buffer containing 20 mM Hepes, pH 7.4, 150 mM NaCl, and 0.01% (w/v) C_12_E_9_. The target protein was eluted with wash buffer plus 5 mM d-desthiobiotin (IBA) and concentrated to a final volume of approximately 100 μL. The final protein was applied to size-exclusion chromatography (Superpose-6 10/300 GL, GE Healthcare) in a buffer containing 20 mM Hepes, pH 7.4, 150 mM NaCl, and 0.01% C_12_E_9_. The peak corresponding to the ABCB6 or ABCB6 mutant was collected and protein concentrations in detergent micelles were measured by absorbance at 280 nm using a NanoDrop (Implen).

### Nanodisc reconstitution

A mixture of lipids containing POPC and POPE (3:1 wt:wt) were solubilized in 1% C_12_E_9_ and mixed with nanodisc scaffold protein MSP2N2 and ABCB6 or mutant protein in a stoichiometry of 150:4:1, incubated for 1 h at 4 °C. Nanodisc formation was induced by removing detergent with Bio-Beads (Bio-Rad) overnight at 4 °C. Samples were concentrated on a 100,000 molecular weight cutoff centrifugal filter. Protein concentrations in the nanodisc were measured by gel densitometry analyzed in ImageJ. The reconstituted ABCB6–MSP complex was used for either ATP-assay or cryo-microscopy data collection.

### ATPase activity assays

The ABCB6 proteins in detergent micelles and nanodisc used for ATPase activity assay were purified as mentioned above. The ATPase activity was measured using the ADP-Glo Max assay (Promega). All reactions were performed using the reaction buffer from the assay kit at a final condition of 50 mM tris, 10 mM MgCl_2_, pH 8.0. Before starting the reaction, protein and substrates were preheated at 37 °C for 5 min. The reactions were carried out for 50 min at 37 °C and were stopped by the addition of the reagent buffer from the assay kit. The next few steps were followed by ADP-Glo™ Max Assay Protocols and measured luminescence with a plate-reading luminometer^53^. ATPase rates were determined using linear and Nonlinear regression to the Michaelis–Menten equation in GraphPad Prism 8. The mutant ATPase rates significantly different were analyzed by unpaired t-test in GraphPad Prism 8.

### Cryo-EM and image processing

The cryo-EM grids were prepared using Vitrobot Mark IV (FEI) operated at 8 °C and 100% humidity. Before cryo-EM sample preparation, the mutant sample is incubated with 5 mM ATP–Mg^2+^ for 30 min at 37 °C. For samples of ABCB6 and mutant nanodisc, 4-μL aliquots of samples at concentrations of approximately 2 mg/mL were applied onto glow-discharged holey carbon grids (Quantifoil R1.2/1.3) 400-mesh Au grid. After a waiting time of 5 s, the grids were blotted for 2 s and plunged into liquid ethane for quick freezing. The cryo-EM grids were screened on a Tecnai Arctica microscope (FEI) operated at 200 kV using a Falcon II 4k × 4k camera (FEI).

The qualified ABCB6 specimens were transferred into a Titan Krios microscope (FEI) operated at 300 kV for data acquisition and the Gatan K3 Summit detector was equipped with a GIF Quantum energy filter. Images were automatically recorded using SerialEM with a slit width of 20 eV on the energy filter and in super-resolution mode at a nominal magnification of 130,000×, corresponding to a calibrated pixel size of 0.86 Å at object scale, and with defocus ranging from 1.5 to 2.5 μm. Each stack was exposed for 3 s with an exposing time of 0.094 s per frame, resulting in a total of 32 frames per stack, and the total dose rate for each stack was about 50 e/Å^2^.

Each micrograph was corrected for subregion motion correction and dose weighting using UCSF MotionCor2^54^. Gctf was used to determine the contrast transfer function (CTF) parameter and produced the CTF power spectrum on the basis of summed micrographs from MotionCor2 for all micrographs^55^. For the dataset of inward-facing conformation, the 4549 CTF-corrected cryo-EM images were manually selected. Particles were auto picked on micrographs with dose-weighting using RELION^56^. Briefly, about 1000 particles were manually picked from a subset of images and extracted in a box size of 200 pixels and a mask diameter of 200 Å. Extracted particles were subjected to 2D classification requesting ten classes, eight classes of which showed representative views and were selected as templates for automated particle picking. The resulting 2D averages served as the templates for particle auto picking, 2720 K particles picked from 4549 images. For the dataset, particle selection, 2D, and 3D classifications were performed on a binned dataset with a pixel size of 3.44 Å using RELION, which was then manually inspected to exclude noise and other bad particles. Two rounds of 2D classification requesting 100 classes resulted in 1450 K particles and two rounds of 3D classification yielded 393,412 particles. An initial model was generated from 20,000 best particles using cryoSPARC ab initio reconstruction requesting five classes with C1 symmetry^57^. A total of 393,412 particles from these 3D classes was re-extracted to the original pixel size of 0.86 Å and classified into five classes with C1 symmetry using a reference model generated from cryoSPARC, which had been low-pass filters to 20 Å. Two rounds of 3D classification and the most populated class containing 259,824 particles were subjected to further 3D auto refinement with C2 symmetry. The refinement resulted in an overall structure at a resolution of 3.87 Å, which allowed initial model building. To further improve the resolution, we performed CTF refinement, which yielded a map at 3.61-Å resolution.

The qualified ABCB6[EQ] specimens were transferred into a Titan Krios microscope (FEI) operated at 300 kV for data acquisition and the Gatan K3 Summit detector. Images were automatically recorded using SerialEM and in super-resolution mode at a nominal magnification of 81,000×, corresponding to a calibrated pixel size of 0.97 Å at object scale, and with defocus ranging from 1.5 to 2.5 μm. Each stack was exposed for 3 s with an exposing time of 0.094 s per frame, resulting in a total of 32 frames per stack, and the total dose rate for each stack was about 50 e/ Å^2^.

For the dataset of ATP-bound conformation, the 3100 K particles performed two rounds of 2D classification and one round of 3D classification that obtained 413,835 good particles. After re-extraction to the original pixel size of 0.97 Å, 218,211 particles were subjected to further 3D auto refinement. The global resolution for hABCB6 occluded conformation with C1 symmetry was 3.66 Å and C2 symmetry was 3.52 Å. All reported resolutions are based on the gold-standard Fourier Shell Correlation (FSC) = 0.143 criteria^58^, and the final FSC curve was corrected for the effect of a soft mask using high-resolution noise substitution. The final density maps were sharpened by B-factors calculated with the RELION post-processing program. The local resolution map was calculated using ResMap^59^ and displayed in Chimera^60^.

### Model building and refinement

Models of full-length hABCB6 were predicted on I-TASSER server^61^. The predicted models were docked into the cryo-EM map with a resolution of 3.6 and 3.5 Å in Chimera and manually adjusted in Coot to acquire the atomic model of hABCB6. Sequence alignment and secondary structure prediction of hABCB6 were used to aid the model building. Model refinement was performed on the main chain of the two atom models using real_space_refine module of PHENIX^62^ with secondary structure and geometry restraints to avoid overfitting. The geometry of the models was evaluated by Molprobity^63^.

### Molecular docking of PPIX and GSH

To obtain a model of the ABCB6–PPIX and ABCB6–GSH structure, we docked the ligands to inward-facing conformation ABCB6 in AutoDock Vina. The protein was used as the receptor and prepared in AutoDockTools. Hydrogen atoms and protein charges were added before the grid file formed. PPIX and GSH were also prepared in AutoDockTools to finish the process of hydrogen atoms and charges adding and root operation. The docking box was centered on the position of cavity 1 besides the plug. The PPIX and GSH were docked into ABCB6 using AutoDock Vina and the output file was presented by ViewDock and dock results were analyzed according to the favorable interaction energy^64^.

## Supplementary information

Supplementary information

## Data Availability

The 3D cryo-EM density maps of occluded and inward-facing hABCB6 have been deposited in the Electron Microscopy Data Bank (EMDB), with accession codes EMD 31169, EMD 31170. The coordinates of atomic models have been deposited in the Protein Data Bank (PDB), with the accession codes 7EKL and 7EKM.

## References

[1] Locher KP (2016). Mechanistic diversity in ATP-binding cassette (ABC) transporters. Nat. Struct. Mol. Biol..

[2] Linton KJ, Higgins CF (1998). The *Escherichia coli* ATP-binding cassette (ABC) proteins. Mol. Microbiol..

[3] Stefkova J, Poledne R, Hubacek JA (2004). ATP-binding cassette (ABC) transporters in human metabolism and diseases. Physiol. Res..

[4] van Veen HW, Konings WN (1997). Multidrug transporters from bacteria to man: similarities in structure and function. Semin. Cancer Biol..

[5] Robey RW (2018). Revisiting the role of ABC transporters in multidrug-resistant cancer. Nat. Rev. Cancer.

[6] Dean M, Rzhetsky A, Allikmets R (2001). The human ATP-binding cassette (ABC) transporter superfamily. Genome Res..

[7] Burke MA, Ardehali H (2007). Mitochondrial ATP-binding cassette proteins. Transl. Res..

[8] Beis K (2015). Structural basis for the mechanism of ABC transporters. Biochem. Soc. Trans..

[9] Boswell-Casteel RC, Fukuda Y, Schuetz JD (2017). ABCB6, an ABC transporter impacting drug response and disease. AAPS J..

[10] Minami K (2014). Expression of ABCB6 is related to resistance to 5-FU, SN-38 and vincristine. Anticancer Res..

[11] Yasui K (2004). Alteration in copy numbers of genes as a mechanism for acquired drug resistance. Cancer Res..

[12] Park S (2006). Gene expression profiling of ATP-binding cassette (ABC) transporters as a predictor of the pathologic response to neoadjuvant chemotherapy in breast cancer patients. Breast Cancer Res. Treat..

[13] Rakvacs Z (2019). The human ABCB6 protein is the functional homologue of HMT-1 proteins mediating cadmium detoxification. Cell. Mol. Life Sci..

[14] Lynch J, Fukuda Y, Krishnamurthy P, Du G, Schuetz JD (2009). Cell survival under stress is enhanced by a mitochondrial ATP-binding cassette transporter that regulates hemoproteins. Cancer Res..

[15] Helias V (2012). ABCB6 is dispensable for erythropoiesis and specifies the new blood group system Langereis. Nat. Genet..

[16] Krishnamurthy P, Schuetz JD (2011). The role of ABCG2 and ABCB6 in porphyrin metabolism and cell survival. Curr. Pharm. Biotechnol..

[17] Krishnamurthy P, Xie T, Schuetz JD (2007). The role of transporters in cellular heme and porphyrin homeostasis. Pharmacol. Ther..

[18] Paterson JK (2007). Human ABCB6 localizes to both the outer mitochondrial membrane and the plasma membrane. Biochemistry.

[19] Krishnamurthy PC (2006). Identification of a mammalian mitochondrial porphyrin transporter. Nature.

[20] Chiabrando D, Vinchi F, Fiorito V, Mercurio S, Tolosano E (2014). Heme in pathophysiology: a matter of scavenging, metabolism and trafficking across cell membranes. Front. Pharmacol..

[21] Ulrich DL (2012). ATP-dependent mitochondrial porphyrin importer ABCB6 protects against phenylhydrazine toxicity. J. Biol. Chem..

[22] Smith AG, Raven EL, Chernova T (2011). The regulatory role of heme in neurons. Metallomics.

[23] Wang C (2020). Cryo-electron microscopy structure of human ABCB6 transporter. Protein Sci..

[24] Higgins CF, Linton KJ (2004). The ATP switch model for ABC transporters. Nat. Struct. Mol. Biol..

[25] Chavan H, Khan MMT, Tegos G, Krishnamurthy P (2013). Efficient purification and reconstitution of ATP binding cassette transporter B6 (ABCB6) for functional and structural studies. J. Biol. Chem..

[26] Haffke M, Menzel A, Carius Y, Jahn D, Heinz DW (2010). Structures of the nucleotide-binding domain of the human ABCB6 transporter and its complexes with nucleotides. Acta Crystallogr. D. Biol. Crystallogr..

[27] Johnson ZL, Chen J (2018). ATP binding enables substrate release from multidrug resistance protein 1. Cell.

[28] Lee KPK, Chen J, MacKinnon R (2017). Molecular structure of human KATP in complex with ATP and ADP. Elife.

[29] Wu JX (2018). Ligand binding and conformational changes of SUR1 subunit in pancreatic ATP-sensitive potassium channels. Protein Cell.

[30] Oldham ML (2016). A mechanism of viral immune evasion revealed by cryo-EM analysis of the TAP transporter. Nature.

[31] Lee JY, Yang JG, Zhitnitsky D, Lewinson O, Rees DC (2014). Structural basis for heavy metal detoxification by an Atm1-type ABC exporter. Science.

[32] Fan C, Kaiser JT, Rees DC (2020). A structural framework for unidirectional transport by a bacterial ABC exporter. Proc. Natl. Acad. Sci. USA.

[33] Taylor NMI (2017). Structure of the human multidrug transporter ABCG2. Nature.

[34] Jackson SM (2018). Structural basis of small-molecule inhibition of human multidrug transporter ABCG2. Nat. Struct. Mol. Biol..

[35] Manolaridis I (2018). Cryo-EM structures of a human ABCG2 mutant trapped in ATP-bound and substrate-bound states. Nature.

[36] Johnson ZL, Chen J (2017). Structural basis of substrate recognition by the multidrug resistance protein MRP1. Cell.

[37] Liu F, Zhang Z, Csanady L, Gadsby DC, Chen J (2017). Molecular structure of the human CFTR ion channel. Cell.

[38] Moody JE, Millen L, Binns D, Hunt JF, Thomas PJ (2002). Cooperative, ATP-dependent association of the nucleotide binding cassettes during the catalytic cycle of ATP-binding cassette transporters. J. Biol. Chem..

[39] Kuhnke G, Neumann K, Muhlenhoff U, Lill R (2006). Stimulation of the ATPase activity of the yeast mitochondrial ABC transporter Atm1p by thiol compounds. Mol. Membr. Biol..

[40] Srinivasan V, Pierik AJ, Lill R (2014). Crystal structures of nucleotide-free and glutathione-bound mitochondrial ABC transporter Atm1. Science.

[41] Zutz A, Gompf S, Schagger H, Tampe R (2009). Mitochondrial ABC proteins in health and disease. Biochim. Biophys. Acta.

[42] Lill R, Kispal G (2001). Mitochondrial ABC transporters. Res. Microbiol..

[43] Fukuda Y (2016). The severity of hereditary porphyria is modulated by the porphyrin exporter and Lan antigen ABCB6. Nat. Commun..

[44] Zhang C (2013). Mutations in ABCB6 cause dyschromatosis universalis hereditaria. J. Invest. Dermatol..

[45] Wang L (2012). ABCB6 mutations cause ocular coloboma. Am. J. Hum. Genet..

[46] Andolfo I (2013). Missense mutations in the ABCB6 transporter cause dominant familial pseudohyperkalemia. Am. J. Hematol..

[47] Bawazir WM (2014). Familial pseudohyperkalemia in blood donors: a novel mutation with implications for transfusion practice. Transfusion.

[48] Cui YX (2013). Novel mutations of ABCB6 associated with autosomal dominant dyschromatosis universalis hereditaria. PLoS One.

[49] Andolfo I (2016). Functional characterization of novel ABCB6 mutations and their clinical implications in familial pseudohyperkalemia. Haematologica.

[50] Zhou S (2005). Increased expression of the Abcg2 transporter during erythroid maturation plays a role in decreasing cellular protoporphyrin IX levels. Blood.

[51] Ward A, Reyes CL, Yu J, Roth CB, Chang G (2007). Flexibility in the ABC transporter MsbA: alternating access with a twist. Proc. Natl. Acad. Sci. USA.

[52] Orlando BJ, Liao MF (2020). ABCG2 transports anticancer drugs via a closed-to-open switch. Nat. Commun..

[53] Zegzouti H, Zdanovskaia M, Hsiao K, Goueli SA (2009). ADP-Glo: a bioluminescent and homogeneous ADP monitoring assay for kinases. Assay Drug Dev. Technol..

[54] Zheng SQ (2017). MotionCor2: anisotropic correction of beam-induced motion for improved cryo-electron microscopy. Nat. Methods.

[55] Zhang K (2016). Gctf: Real-time CTF determination and correction. J. Struct. Biol..

[56] Scheres SH (2012). RELION: implementation of a Bayesian approach to cryo-EM structure determination. J. Struct. Biol..

[57] Punjani A, Rubinstein JL, Fleet DJ, Brubaker MA (2017). cryoSPARC: algorithms for rapid unsupervised cryo-EM structure determination. Nat. Methods.

[58] Scheres SH, Chen S (2012). Prevention of overfitting in cryo-EM structure determination. Nat. Methods.

[59] Swint-Kruse L, Brown CS (2005). Resmap: automated representation of macromolecular interfaces as two-dimensional networks. Bioinformatics.

[60] Pettersen EF (2004). UCSF Chimera―a visualization system for exploratory research and analysis. J. Comput. Chem..

[61] Zhang Y (2008). I-TASSER server for protein 3D structure prediction. BMC Bioinforma..

[62] Adams, P. D. et al. PHENIX: a comprehensive Python-based system for macromolecular structure solution. *Acta Crystallogr. D. Biol. Crystallogr.* **66**, 213–221 (2010). 10.1107/S0907444909052925 PMC2815670 20124702

[63] Chen, V. B. et al. MolProbity: all-atom structure validation for macromolecular crystallography. *Acta Crystallogr. D. Biol. Crystallogr.* **66**, 12–21 (2010). 10.1107/S0907444909042073 PMC2803126 20057044

[64] Trott O, Olson AJ (2010). AutoDock Vina: improving the speed and accuracy of docking with a new scoring function, efficient optimization, and multithreading. J. Comput. Chem..

