# Supplementary material for: Molecular insights into the human ABCB6 transporter
Source: Cell Discov. 2021 Jul 27;7:55. doi: 10.1038/s41421-021-00284-z (PMC8313675; doi:10.1038/s41421-021-00284-z)
Supplement: Supplementary file 1 — Supplementary information [file 41421_2021_284_MOESM1_ESM.pdf]

Supplementary Materials for

Molecular insights into the human ABCB6 transporter

Guangyuan Song[1*], Sensen Zhang[1*], Mengqi Tian[1*], Laixing Zhang[1*], Runyu Guo[1], Wei Zhuo[1], Maojun Yang[1#]

[1]*Ministry of Education Key Laboratory of Protein Science, Tsinghua-Peking Center for Life Sciences, Beijing Advanced Innovation Center for Structural Biology, School of Life Sciences, Tsinghua University, Beijing 100084, China.*

[*]These authors contribute equally to this work.

[#]To whom correspondence should be addressed.

This file includes:

Supplementary Figs. S1 to S11

Supplementary Table S1

Supplementary Figures and Figure legends

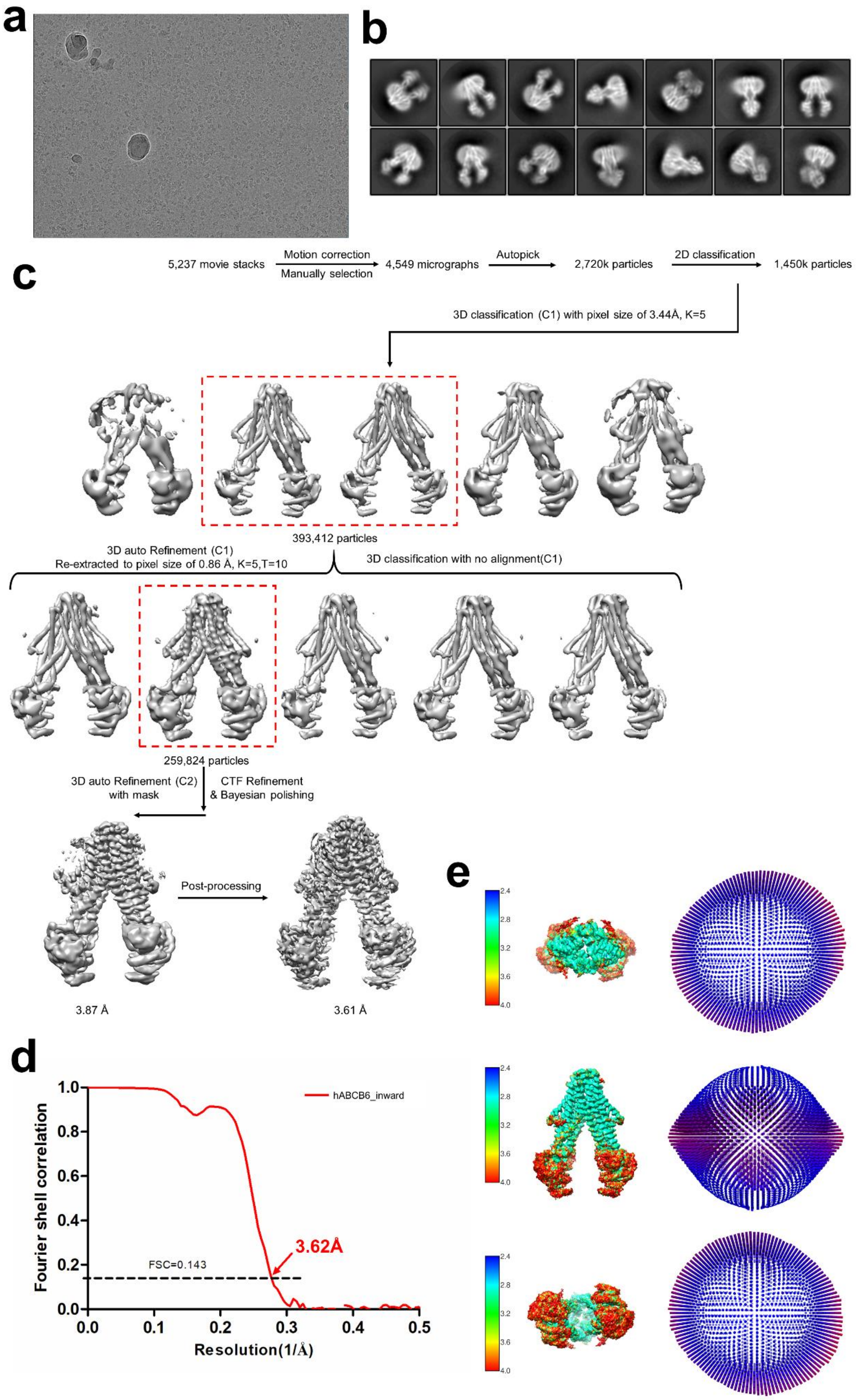

Supplementary Fig. S1 Reconstruction and structure determination of the apo-state hABCB6.

a, Representative cryo-EM micrograph of apo-state hABCB6 in nanodisc.

b, 2D class averages of the apo-state hABCB6 sample in nanodisc.

c, The workflow of 2D/3D reconstruction with apo-state hABCB6 cryo-EM data. In brief, 1,450 k particles were kept after 2D classification, and subjected to three rounds of 3D classification. A final dataset containing 260 k particles were used for high-resolution refinement (see methods for more details).

d, Gold-standard Fourier Shell correlation (FSC) curve of apo state hABCB6 after 3D refinement. The resolution estimation was based on the criterion of FSC 0.143 cutoff.

e, Angular distribution of the apo-state hABCB6 final reconstruction.

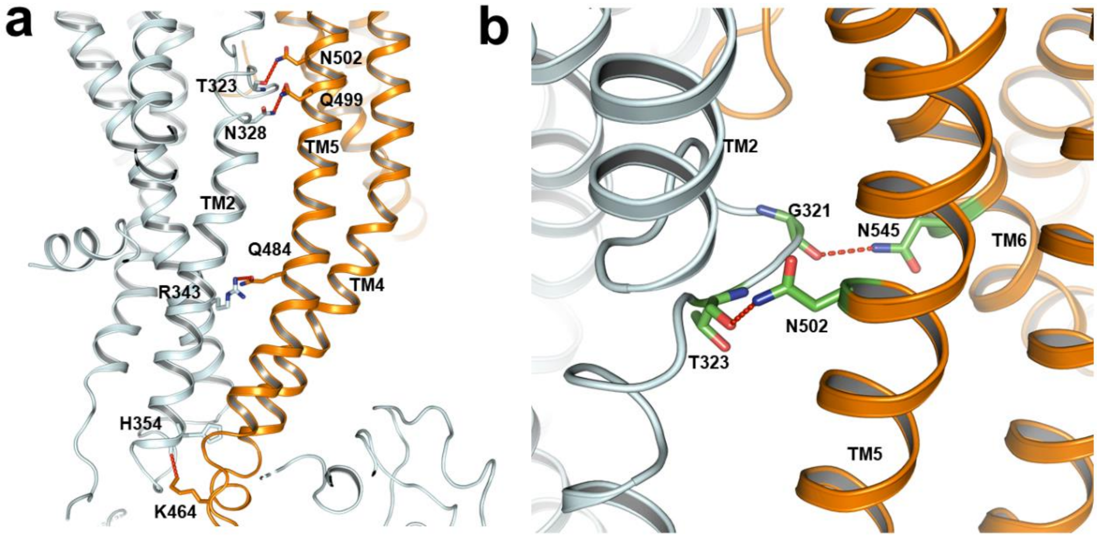

Supplementary Fig. S2 The interactions within two hABCB6 monomers.

a, The interaction between TM2 and TM5 from two neighboring monomers.

b, The interaction between the “plug” and TM6 from two neighboring monomers.

a

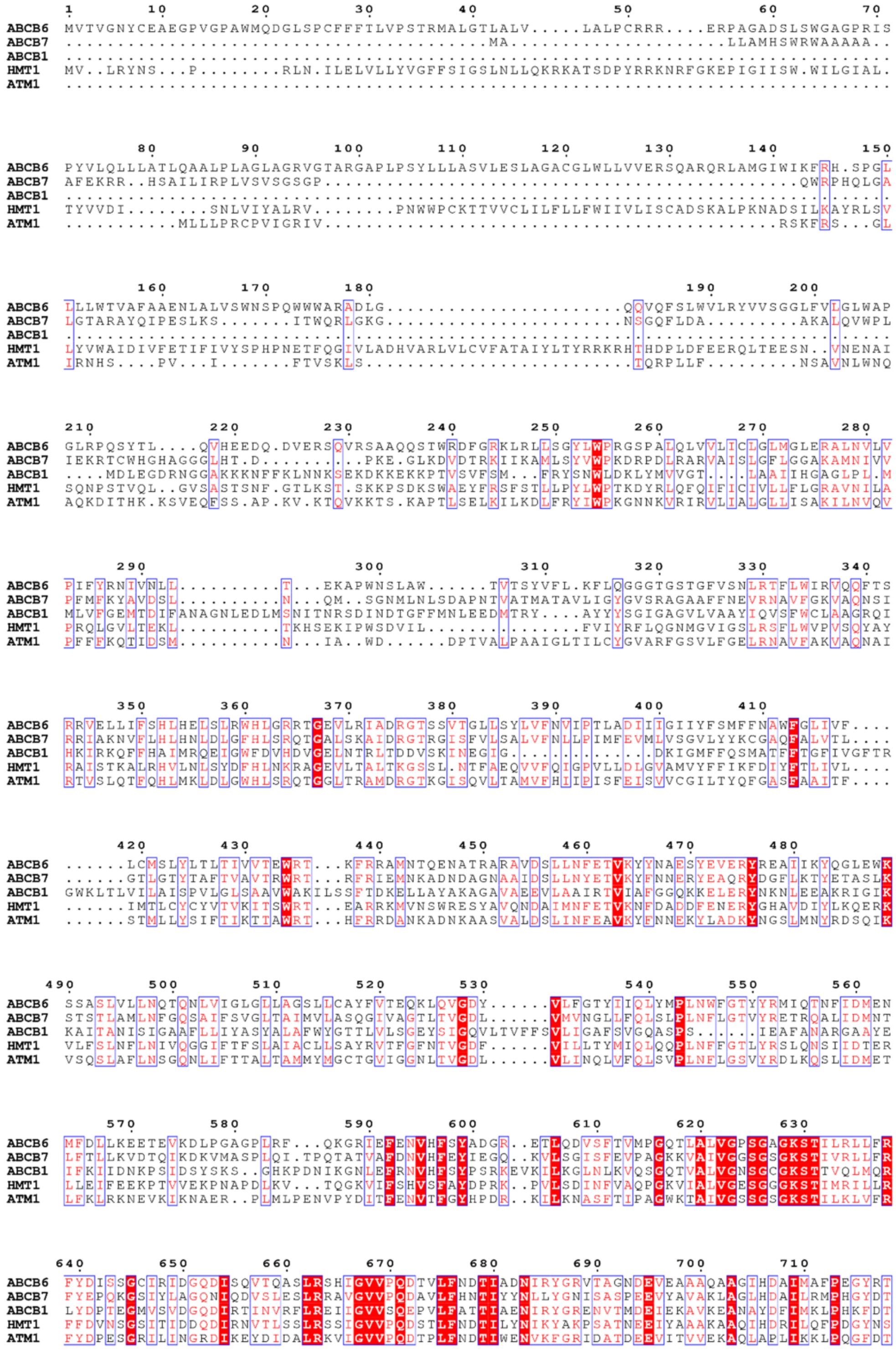

b

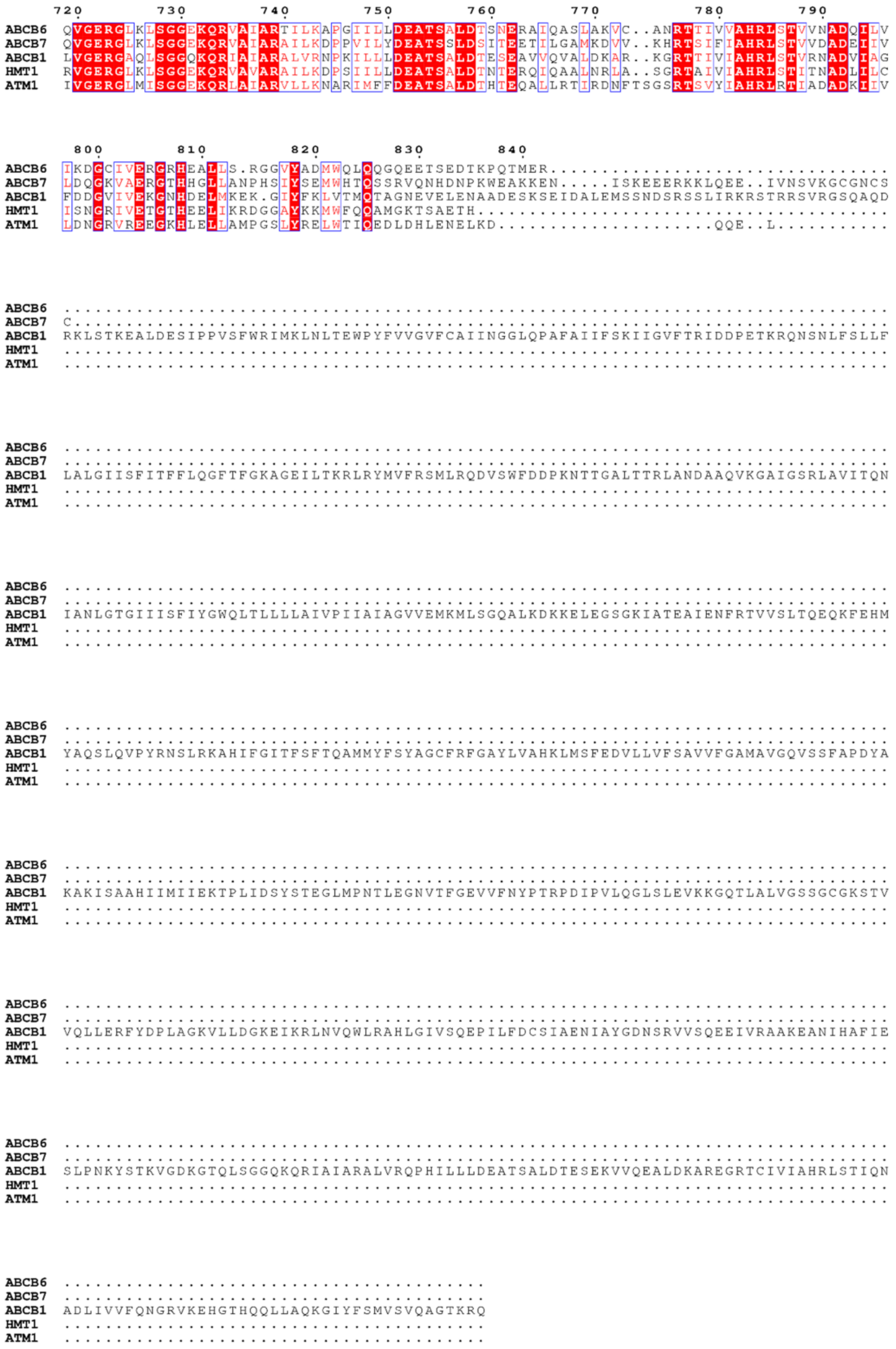

Supplementary Fig. S3 Sequence alignment of ABC transporters.

a,b, Sequence alignment of human ABCB1, ABCB6, ABCB7 and yeast HMT1, ATM1 using ESPript3.

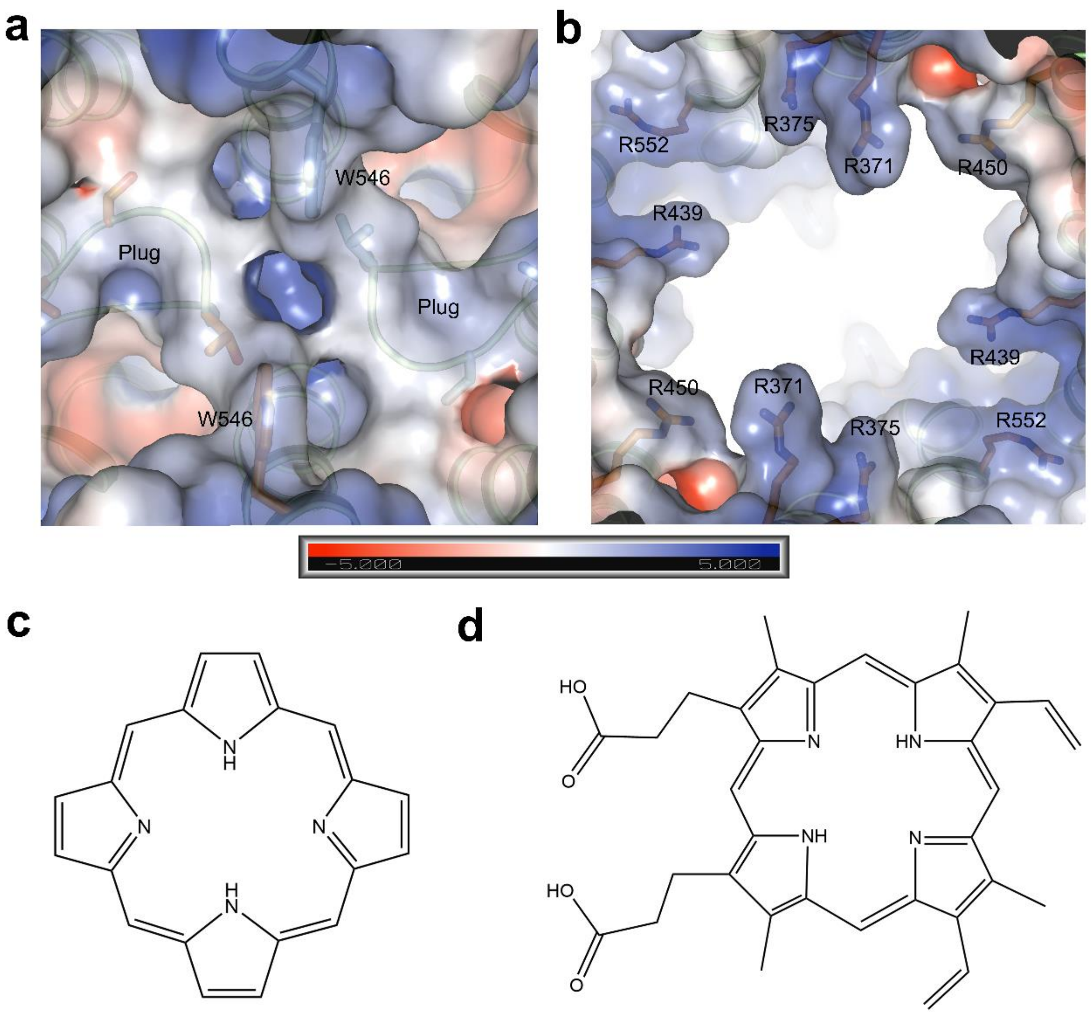

porphyrin **Protoporphyrin IX**

Supplementary Fig. S4 The H-P pocket of hABCB6.

a,b, The electrostatic properties of the interior surface of the translocation pathway. hydrophobic region (a) and positive region (b) were shown separately. Scale: red, negative (-5 kT/e); blue, positive (+5 kT/e). Two “plugs” and W546 are shown in (a). Side chains of representative arginine are shown in (b).

c, The structure of porphyrin.

d, The structure of protoporphyrin IX.

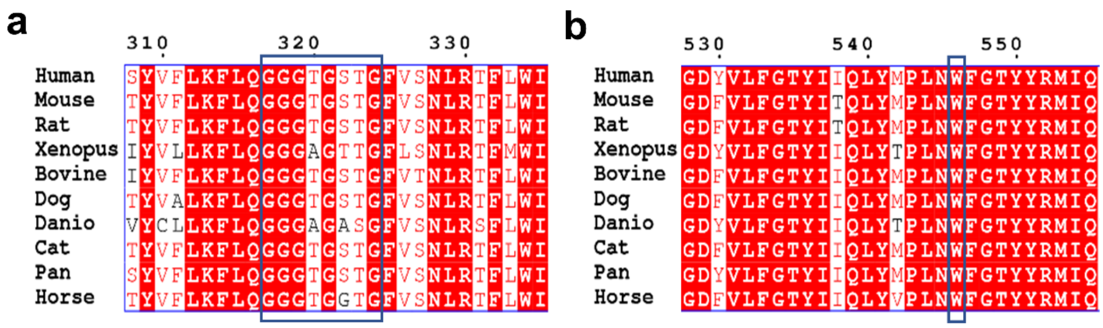

Supplementary Fig. S5 Sequence alignment of the "plug" and W546.

a, Sequence alignment of the "plug" in ABCB6 among different species.

b, Sequence alignment of W546 in ABCB6 among different species.

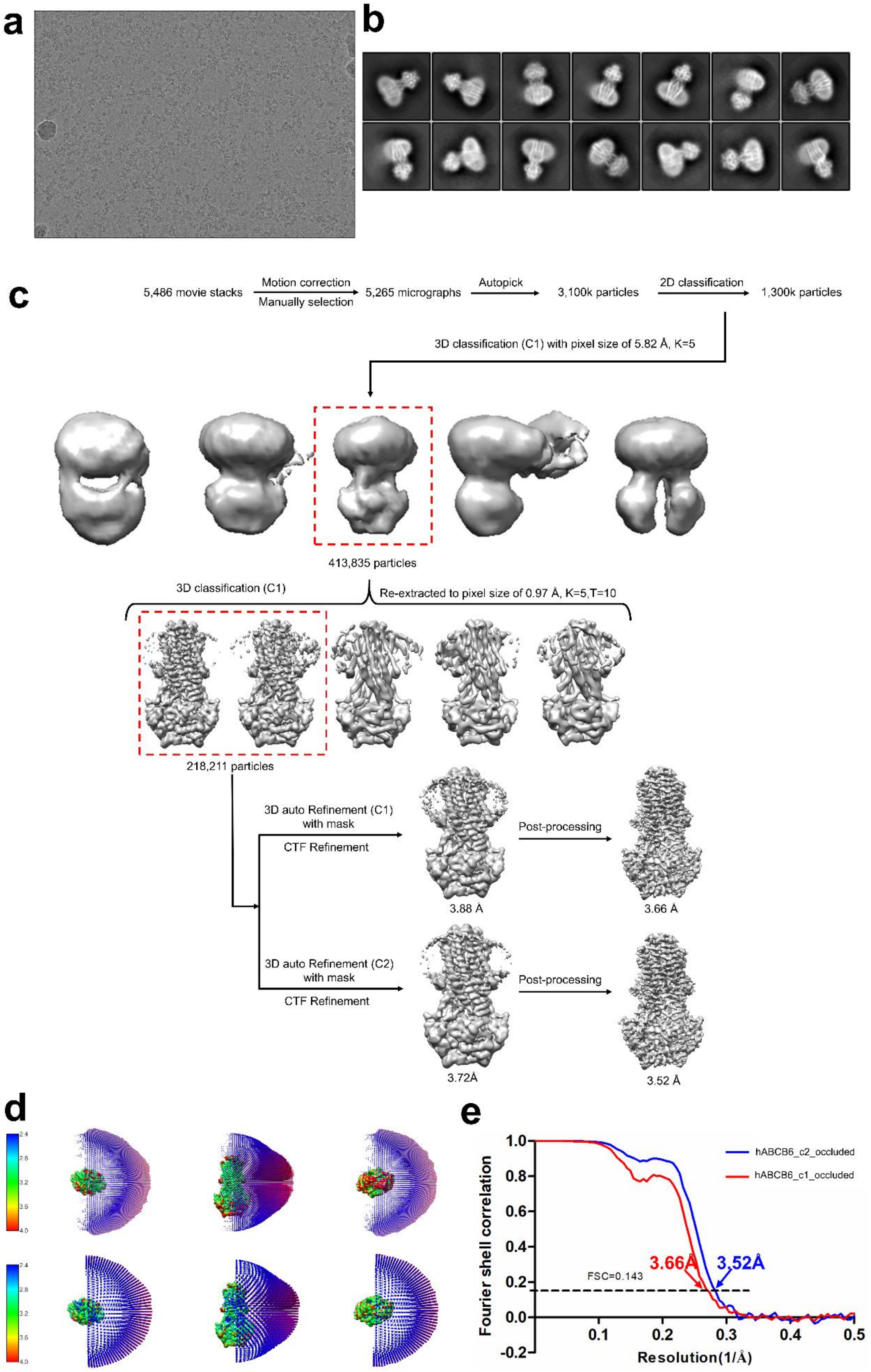
a
b
c
5,486 movie stacks
Motion correction
Manually selection
5,265 micrographs
Autopick
3,100k particles
2D classification
1,300k particles
3D classification (C1) with pixel size of 5.82 Å, K=5
413,835 particles
3D classification (C1)
Re-extracted to pixel size of 0.97 Å, K=5,T=10
218,211 particles
3D auto Refinement (C1)
with mask
CTF Refinement
Post-processing
3.88 Å
3.66 Å
3D auto Refinement (C2)
with mask
CTF Refinement
Post-processing
3.72Å
3.52 Å
d
e
Fourier shell correlation
Resolution(1/Å)
hABCB6_c2_occluded
hABCB6_c1_occluded
FSC=0.143
3.66Å
3.52Å

Supplementary Fig. S6 Reconstruction and structure determination of the nucleotide-bound hABCB6.

a, Representative cryo-EM micrograph of nucleotide-bound state hABCB6 in nanodisc.

b, 2D class averages of the nucleotide-bound state hABCB6 sample in nanodisc.

c, The workflow of 2D/3D reconstruction with nucleotide-bound state hABCB6 cryo-EM data. In brief, 1,300 k particles were kept after 2D classification, and subjected to three rounds of 3D classification. A final dataset containing 218 k particles were used for high-resolution refinement (see methods for more details).

d, Angular distribution of the nucleotide-bound state hABCB6 final reconstruction.

e, Gold-standard Fourier Shell correlation (FSC) curve of nucleotide-bound state hABCB6 after 3D refinement. The resolution estimation was based on the criterion of FSC 0.143 cutoff.

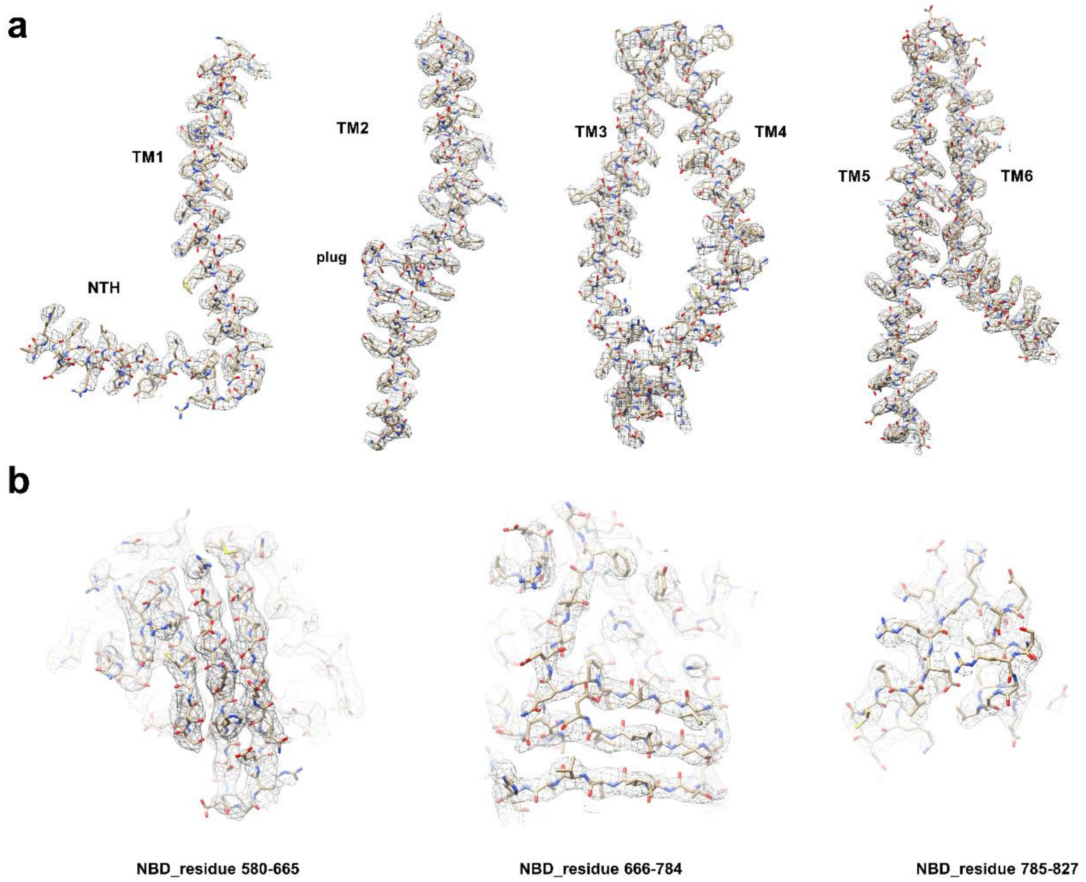

Supplementary Fig. S7 Representative cryo-EM densities of hABCB6.

a, Density maps of representative transmembrane regions of nucleotide-bound hABCB6. Stick style atomic models (gold) were fitted into the cryo-EM density maps (gray mesh). The density maps were contoured at 10.0 σ.

b, Density maps of the representative nucleotide-binding region of nucleotide-bound hABCB6, similar to the panel (a).

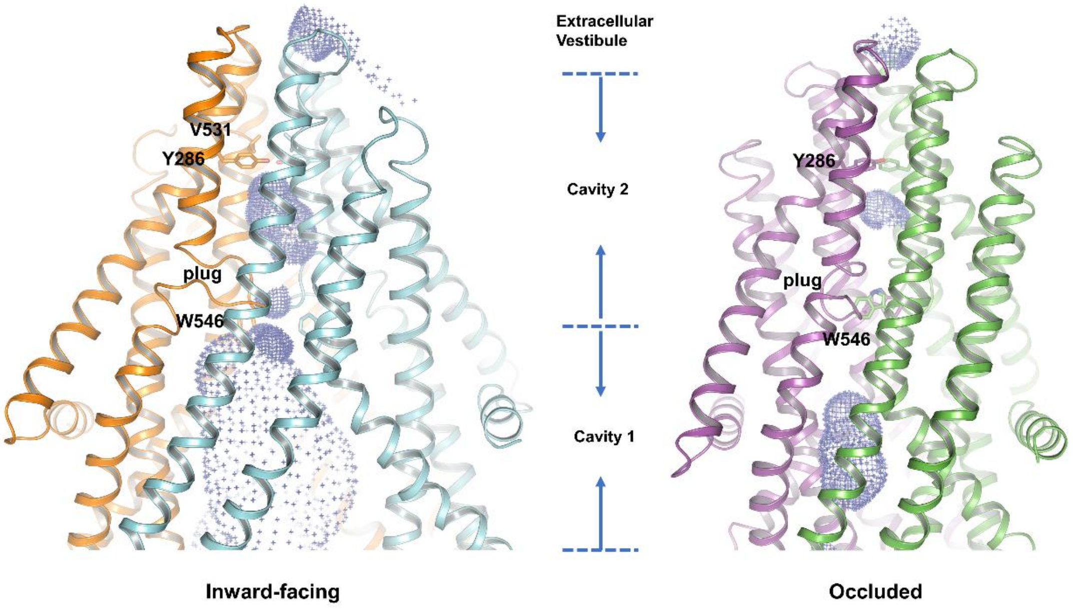

Supplementary Fig. S8 The translocation pathway in hABCB6.

The substrate translocation pathway of hABCB6 in two conformations calculated by HOLE software. The pockets are shown as meshed surface and two cavities are divided by the plug or W546 in different conformations.

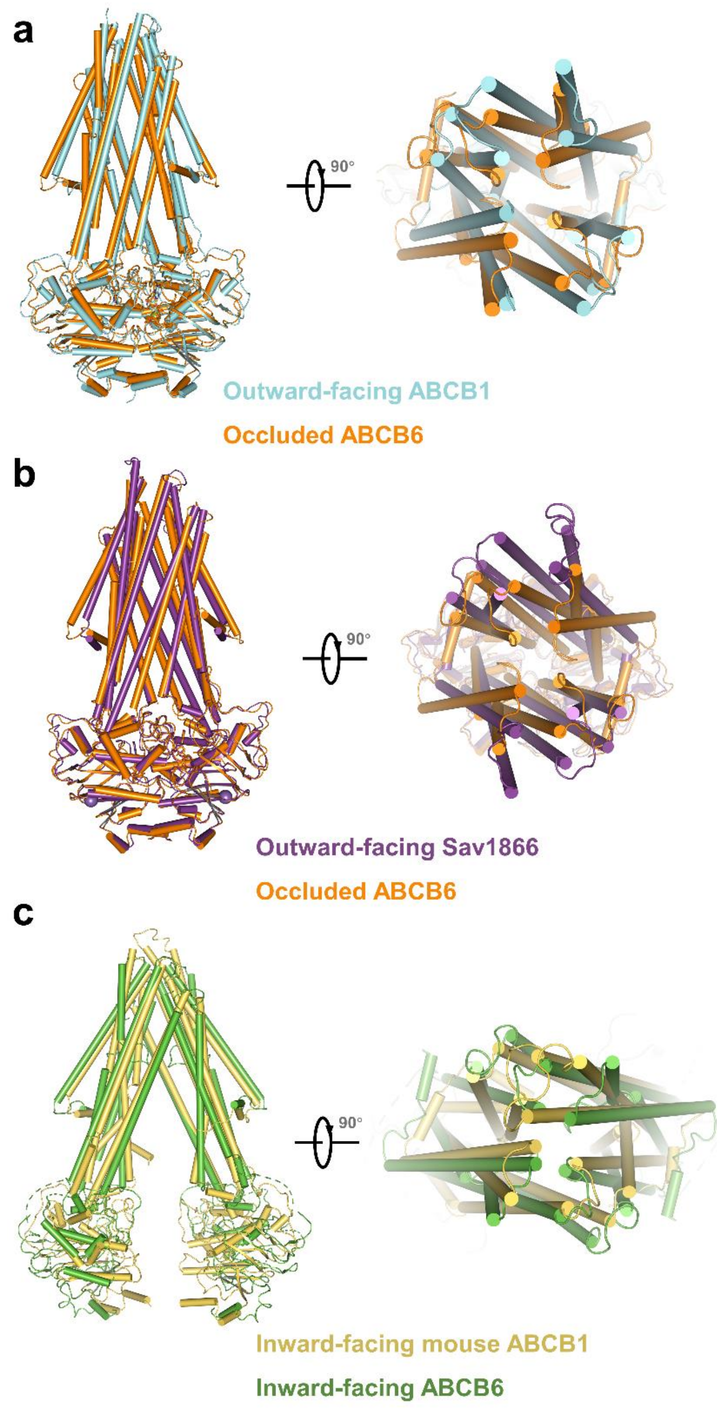

Supplementary Fig. S9 Comparisons of hABCB6 with other homologous proteins.

a, Comparison of occluded hABCB6 with outward-facing ABCB1 (6C0V).

b, Comparison of occluded hABCB6 with outward-facing Sav1866 (2HYD).

c, Comparison of inward-facing conformation hABCB6 with mouse ABCB1 (4M1M).

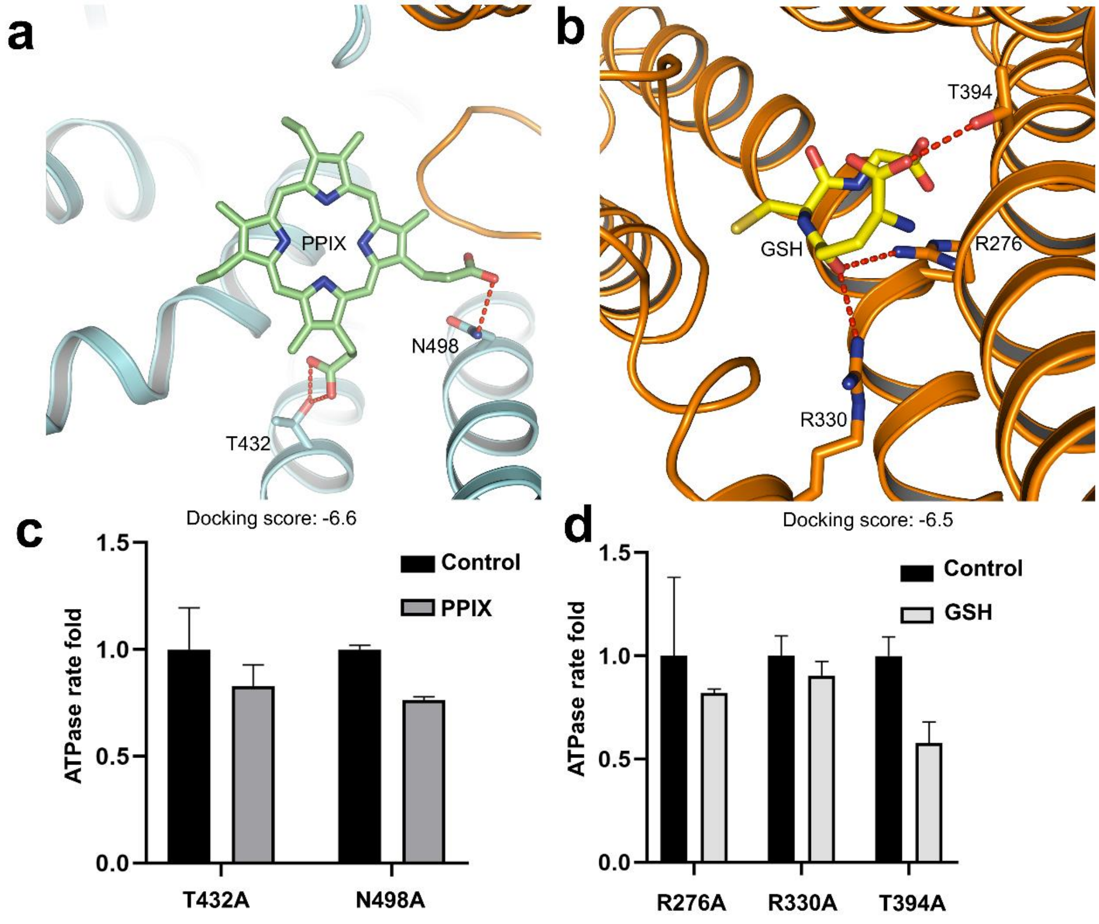

Supplementary Fig. S10 Molecular docking of PPIX and GSH in hABCB6

a, The hABCB6-PPIX dock model and the interactions between PPIX and hABCB6.

b, The hABCB6-GSH dock model and the interactions between GSH and hABCB6.

c, The ATPase rate assay of PPIX-related mutants: T432A and N498A.

d, The ATPase rate assay of GSH-related mutants: R276A, R330A, and T394A.

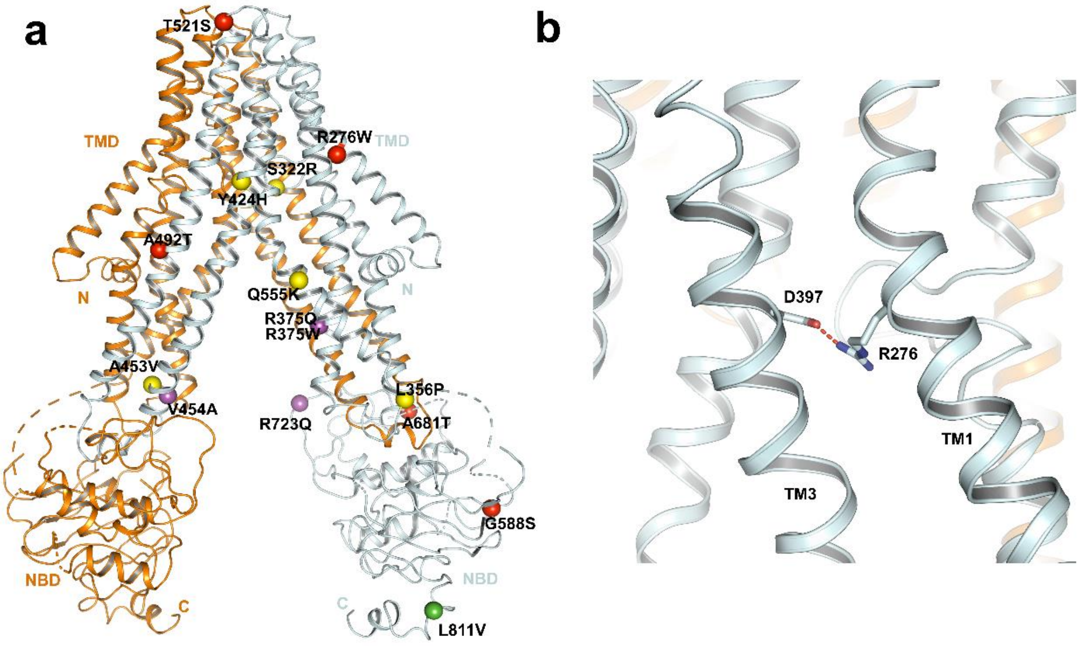

Supplementary Fig. S11 Structural interpretation of the pathogenic mutations.

a, The pathogenic mutations of hABCB6. Mutations related to different diseases are marked with different colors. Red: Porphyria. Yellow: dyschromatosis universalis hereditaria (DUH). Magenta: familial pseudohyperkalemia (FP). Green: ocular coloboma.

b, The interaction between R276 and D397.

Supplementary Table S1 | Cryo-EM data collection, refinement and validation statistics

	ABCB6	ABCB6[EQ]
**Data collection and processing**		
Magnification	130,000	81,000
Voltage (kV)	300	300
Electron exposure ($e^-/Å^2$)	50	50
Defocus range (μm)	-1.5 ~ -2.5	-1.5 ~ -2.5
Pixel size (Å)	0.86	0.97
Software	RELION-3	RELION-3
Symmetry imposed	C2	C2
Initial particle images (no.)	1450,000	1300,000
Final particles images (no.)	259,000	218,000
Map resolution (Å)	3.62	3.52
FSC threshold	0.143	0.143
Local map resolution range (Å)	2.4-4.0	2.4-4.0
**Refinement**		
Software	PHENIX 1.14	PHENIX 1.14
Initial model used (PDB code)		
Model resolution (Å)	3.7	3.6
FSC threshold	0.5	0.5
Map sharpening *B* factor	-162.9	-198.7
Model composition		
Non-hydrogen atoms	8828	9394
Protein residues	782	1184
Ligand	0	0
B factors ($Å^2$)		
Protein	55.36	45.08
Ligand	0	0
R.m.s deviations		
Bond length (Å)	0.005	0.004
Bond angles (°)	1.199	0.839
Validation		
MolProbity score	2.55	1.52
Clashscore	5.69	4.66
Poor rotamers (%)	7.73	0.40
Ramachandran plot		
Favored (%)	89.28	95.92
Allowed (%)	9.56	4.08
Disallowed (%)	0.18	0.00
